# Evaluation of topical erythropoietin application on the healing outcome of gingival graft recipient site; a randomized controlled clinical trial

**DOI:** 10.1186/s12903-021-01948-8

**Published:** 2021-11-12

**Authors:** Siamak Yaghobee, Nina Rouzmeh, Mina Taheri, Hoori Aslroosta, Sanaz Mahmoodi, Masoomeh Mohammadnejad Hardoroodi, Pardis Soleimanzadeh Azar, Afshin Khorsand

**Affiliations:** 1 Periodontics Department, School of Dentistry, Tehran University of Medical Sciences, Tehran, Iran; 2 Independent Researcher, Novin Formula Consulting Group, Tehran, Iran; 3 Private Office, Qaemshahr, Iran; 4 Endodontic Department, UTHSCSA, San Antonio, Texas United States

**Keywords:** Erythropoietin, Wound healing, Gingiva, Inflammation

## Abstract

**Background:**

Free gingival graft (FGG) is a highly predictable method to increase the width of keratinized gingiva. Various materials have been reported to accelerate the wound healing process. Considering the positive effect of EPO on dermal wound healing this study aimed to investigate the effects of EPO on the rate of healing and degree of inflammation in free gingival grafts.

**Methods:**

Seventeen patients with bilateral lack of keratinized gingiva in mandible were selected for this clinical trial. The surgical intervention was performed after phase I periodontal therapy.

Recipient site was prepared apical to the mucogingival line, and FGG was harvested from the palate. Before graft placement, the test side and control side were treated with 1 ml of EPO 4000 IU/ml and distilled water, respectively, for 2 min. On days 7, 14, 21, 28, 60, and 90 after surgery, the grafted sites were examined by blinded observers to compare the healing and inflammation of the areas.

**Results:**

All the 17 patients completed the surgeries and follow-up examinations. Direct examination revealed significantly better healing in EPO group only on the 28th day. Assessment of the photographs showed a significant value in favor of the test group at some other time points as well. The EPO group demonstrated less inflammation, which was statistically significant in many time points. The graft area was 80.88 ± 30.21 mm^2^ and 71.35 ± 15.62 mm^2^ in the EPO and control groups, respectively. The difference was not significant, though.

**Conclusions:**

Topical application of erythropoietin can accelerate the healing of gingival grafts and reduce the inflammation during healing period. The final graft outcome, nevertheless, does not seem to be influenced by EPO.

*Trial registration* This was a split-mouth randomized controlled clinical trial (IRCT201201278830N1). The first registration date: 2016-10-22

## Introduction

Free gingival graft (FGG) is a relatively common procedure among periodontal surgeries. FGG is usually performed to gain wider keratinized gingiva [1], increased vestibular depth [2] and root coverage in recession areas [3]. During FGG technique, a piece of keratinized gingiva (consisting of epithelial and connective tissue) is harvested from the donor site (usually the palate) and placed on an already prepared recipient site [2]. The procedure is reported to have high predictability and effectiveness to achieve desired outcomes [3]. The success of FGG is the result of a complex healing process. Various factors affect the outcome of FGG, among which are case selection and proper application of this technique [4].

According to the histological study by Oliver et al. [5], the healing following free gingival graft can be divided into three phases: initial, revascularization, and maturation. Cytokines and growth factors are essential for adequate healing [6] via the stimulation of angiogenesis, and migration, proliferation, and differentiation of cells.

Traditionally, various materials with mechanical protection, hemostatic and bacteriostatic properties are used to protect the graft in the early stages of graft healing [7]. Eugenol and non-eugenol dressings, along with adhesive barriers such as cyanoacrylate are among those materials [8]. Moreover, various materials have been utilized to accelerate the healing process of periodontal tissues such as Hyaluronic acid which is employed in cell migration and differentiation [9]. In 2013 Briguglio et al. [10] applied hyaluronic acid in intrabony defects and showed that it could effectively support periodontal wound healing.

Curro et al. [11] also revealed the key role of transglutaminases which are expressed in stratified squamous epithelia in gingival remodeling/healing and adaptive processes. In addition, measures such as ozone therapy and low-level laser therapy have been taken to improve healing, with positive results [12, 13].

Erythropoietin (EPO) is a glycoprotein (molecular weight: 30.4 kDa) essential for hematopoiesis and a member of Class I Cytokines [15]. Apart from the hematopoietic effects of this growth factor, EPO is considered a pleiotropic factor and in multiple tissues and organs it has cytoprotective and anti-apoptosis effects [16]. The receptors for EPO have been detected on skin cells such as fibroblasts [17], macrophages [18], mast cells [19] melanocyte [20] and hair follicles [21]. It is shown that EPO enhances the dermal wound healing process by accelerating angiogenesis [22], granulation tissue formation [23], and collagen formation [22]. Animal studies have shown the healing effect of EPO in dermal diabetic lesions or ischemic lesions when applied topically [24, 25] or administered by systemic means [26, 27].

The basal cells of the oral mucosa also express receptors for erythropoietin [28]. Thus, it is expected that topical or systemic application of EPO will be effective on healing of oral lesions. Yaghobee et al. [29] have demonstrated the positive effects of topical application of EPO on surgical palatal wound healing. Furthermore, Hosseinjani et al. [30] have investigated the effects of EPO mouthwash (50 IU/ml, 15 ml four times a day) in the healing of oral mucositis in a randomized controlled trial. Subjects of the study were patients with a history of high-dose chemotherapy after hematopoietic stem cell transplantation. The results revealed that EPO mouthwash could decrease the incidence and duration of mucositis.

In the present study, the effects of topical application of EPO on clinical healing of FGG recipient site have been investigated.

## Materials and methods

This was a split-mouth randomized controlled clinical trial (IRCT201201278830N1). The study protocol was in accordance with the Helsinki Declaration of 1975, revised in 2013. The study was approved by the ethical committee of Tehran University of Medical Sciences (119225).

All study cases were selected from the patients who were referred to the periodontics department, Tehran University of Medical Sciences. The inclusion criteria were: (1) age of 18 years old or above; (2) periodontal health; and (3) need for gingival graft in order to augment keratinized gingiva in at least two mandibular areas. If any of the following criteria were present, the patient would be excluded: (1) compromised systemic health, such as diabetes mellitus and immune suppression; (2) consumption of anti-inflammatory or antibiotics during last 3 months; (3) smoking, and (4) O’Leary plaque index >15% after phase I periodontal therapy. Phase I periodontal treatment, including oral hygiene instructions, scaling, root planing and prophylaxis, if necessary, was performed for the patients meeting the inclusion criteria. Subjects compliant with oral hygiene measures who reached the O’Leary plaque index of less than 15% in the re-evaluation session were selected for the study. Informed consent was obtained from the patients who agreed to participate in the study.

### Sample size calculation

To determine the sample size, using tab analysis of PASS 11, α was set as 0.05 and β was set as 0.2. The clinically relevant difference between the test and control groups was considered, and according to the standard deviation from the study by Yaghobee et al., the sample size was calculated as 17 in each group.

### Randomization and blinding

Balanced block randomization was used to allocate each patient into one of the following categories:

- Right side surgery first, treated with distilled water.
- Right side surgery first, treated with EPO.
- Left side surgery first, treated with distilled water.
- Left side surgery first, treated with EPO..

The patient, the surgeon, the first and last examiner, and the data analyzer were blinded to the intervention.

### Surgical procedure

The classic technique was used for free gingival graft [31]. Surgical graft for both sides of each patient was performed during one session. An assistant opened the sealed envelope to instruct the surgeon to start from the right or left mouth side. Local anesthesia was obtained by local infiltration of lidocaine hydrochloride 2% with 1/80,000 epinephrine (Persocaine-E, Darou Pakhsh Co., Iran). To prepare the bed, a split thickness horizontal incision at mucogingival junction (15 mm long) and two vertical incisions at both ends (10 mm long) were made with scalpel blade no. 15. All tissue tags were removed with scissors to prepare a stable bed with no mobile tissues. A wet gauze was placed on the recipient site to avoid clot formation during palatal graft harvesting. The area of ipsilateral second premolar and first molar was selected for harvesting palatal gingiva. A sterile paper template, prepared exactly the same size and shape of the bed, was used to harvest a piece of palatal gingiva to accommodate the recipient site. The gauze was removed from the bed. At this time, the assistant gave the surgeon the drug or the placebo, according to the randomization envelope. The surgeon remained blinded to the intervention. Test sides were treated with 1 ml erythropoietin (EPO) 4000 IU (PDpoetin, Pooyeshdarou, Tehran, Iran) and the control sides received 1 ml of distilled water. After two minutes, the harvested gingival graft was adapted on the site and stabilized with synthetic absorbable 4/0 sutures (Polyglycolate coated; SUPABON, SUPA, Iran). The graft was then covered with surgical dressing (Coe-Pak; GC America Co., USA).

Analgesics (Gelofen 400 mg, q6h, for 4 days) and antibiotics (amoxicillin 500 mg, q8h, for 7 days) were prescribed for all patients. Routine post-surgical instructions were given to patients in a pamphlet. The surgical dressing was removed 7 days after surgery, and the sutures were removed at 14th day. Professional tooth cleaning and prophylaxis were provided for patients every 2 weeks for 12 weeks.

### Clinical measurements

To evaluate the healing and the inflammation of the surgical site, direct (by one observer) and indirect (by 3 observers through photographs) examinations were performed. For direct observation patients were visited 7, 14, 21, 28, 60, and 90 days after surgery. Each day, one examiner (N. R.), blinded to the test and control sides, compared both surgical sites in each patient in terms of healing status and inflammation severity by direct observation. A standard clinical photograph was also taken from each site for indirect observation by 3 other observers. The photographs were always taken by one camera (Canon SX210 IS) in a specific dental chair at about 10 AM each day, with the chair light off, and at the distance of 30 cm from the surgical site. To compare the test and control sides, two photos were placed side by side in one slide using PowerPoint (Office 2013, Microsoft Co., USA). The magnification values of the photographs were similar. Observers of the photographs were blind to the allocated intervention.

Degree of healing was assessed based on the graft color match, contour, and texture. The inflammation status was evaluated according to the size of the red inflammatory halo around the graft. Direct and indirect examiners decided which side had better conditions, if different at all. At the last visit, the graft dimensions (width and length) were also measured in millimeters through a periodontal probe (University of Michigan “O” probe, Williams markings).

### Statistical analysis

All statistical analysis was performed using SPSS software (IBM SPSS Statistics 20.0). The mean graft dimensions in two groups were compared using Student t-test. Sign test was used to statistically compare the healing and inflammation results in the test and control groups. A p-value of smaller than 0.05 was considered significant difference between the groups.

## Results

**Fig. 1:**
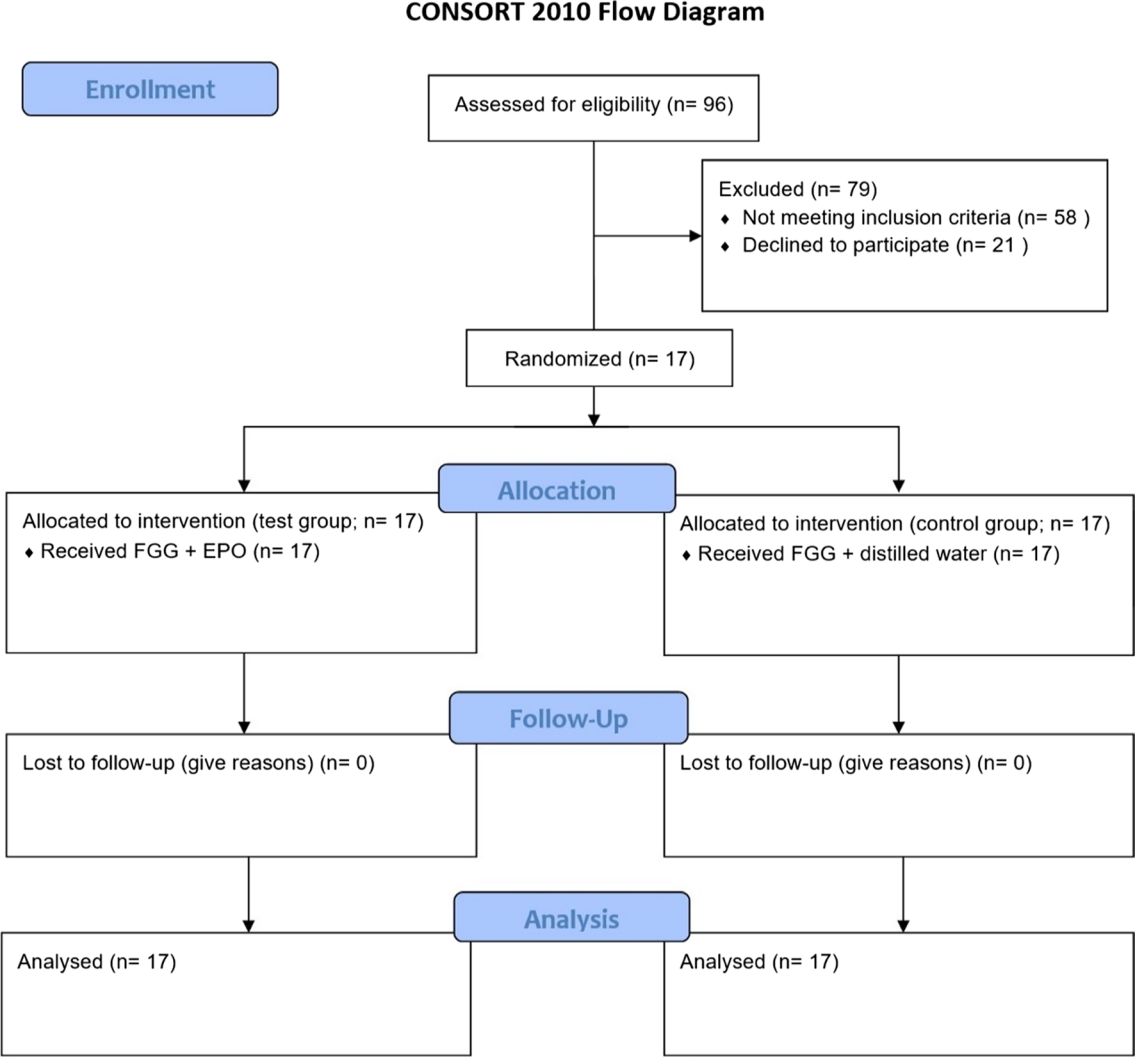
Consort flow diagram

Seventeen patients (5 male, 12 Female) with the mean age of 45 ± 6 years completed the study. Figure 1 shows the consort flow diagram.

**Fig. 2:**
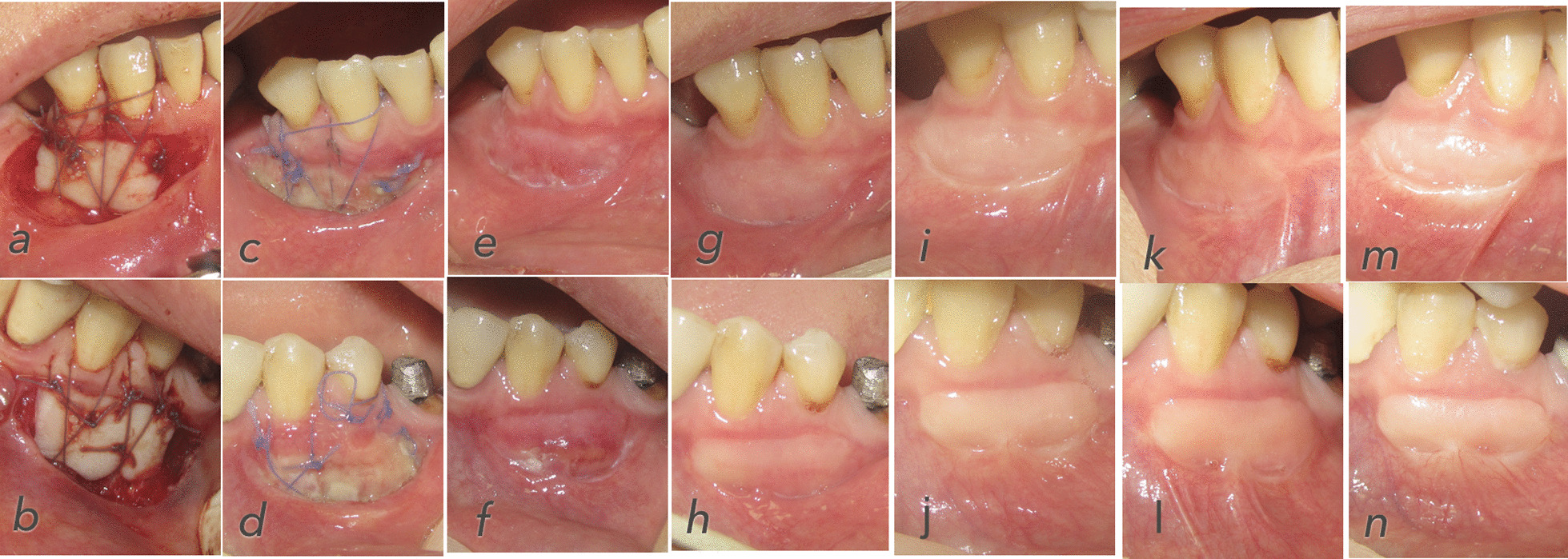
**a** Baseline FGG treatment EPO group, **b** baseline FGG treatment control group, **c** 7 days postop EPO group, **d** 7 days postop control group, **e** 14 days postop EPO group, **f** 14 days postop control group, **g** 21 days postop EPO group, **h** 21 days postop control group, **i** 28 days postop EPO group, **j** 28 days postop control group, **k** 60 days postop EPO group, **l** 60 days postop control group, **m** 90 days postop EPO group, **n** 90 days postop control group

No adverse effect was seen related to the surgery. The clinical photographs of one patient are shown in Fig. 2.

### Healing of soft tissue graft

The results of the evaluation of healing by direct examination and observing the photographs are presented in Table 1. Direct assessment revealed statistically significant difference only on day 28, with 58.8% of the patients showing better results on test side compared to the control side. On the 2nd and 3rd month evaluations, no significant difference was observed by direct or indirect evaluations.

**Table 1:** Healing rate in test vs. control group by direct and indirect evaluation

Healing	Day	EPO > Control	EPO = Control	EPO < Control	p-value
Observer 1	7	5	7	5	1.00
	14	10	3	4	0.11
	21	11	3	3	0.030*
	28	9	4	4	0.260
	60	8	5	4	0.260
	90	9	5	3	0.080
Observer 2	7	12	3	2	0.004*
	14	14	3	0	0.000*
	21	12	2	3	0.010*
	28	10	5	2	0.020*
	60	6	8	3	0.330
	90	8	7	2	0.060
Observer 3	7	6	6	5	0.770
	14	10	3	4	0.110
	21	13	1	3	0.008*
	28	11	4	2	0.008*
	60	6	9	2	0.160
	90	3	10	4	0.720
Direct examination	7	5	5	7	0.580
	14	9	2	6	0.460
	21	7	6	4	0.380
	28	10	4	3	0.050*
	60	9	3	5	0.420
	90	7	4	6	0.790

*significant

### Degree of inflammation

Table 2 shows the results of inflammation assessment by direct and indirect observation of the graft area. The EPO group has normally shown to be better than or equal to the control group, yet direct examination rendered no significant difference in any of the time points.

**Table 2:** Degree of inflammation in test vs. control group by direct and indirect evaluation

Healing	Day	EPO < Control	EPO = Control	EPO > Control	p-value
Observer 1	7	3	8	6	0.330
	14	8	5	4	0.260
	21	7	7	3	0.220
	28	6	8	3	0.330
	60	4	12	1	0.190
	90	2	15	0	0.160
Observer 2	7	7	5	5	0.580
	14	8	2	7	0.810
	21	10	4	3	0.050*
	28	10	6	1	0.003*
	60	8	8	1	0.010*
	90	5	12	0	0.020*
Observer 3	7	6	4	7	0.790
	14	11	4	2	0.008*
	21	14	1	2	0.001*
	28	12	2	3	0.020*
	60	7	7	3	0.060
	90	9	8	0	0.001*
Direct examination	7	5	7	5	1.000
	14	6	8	3	0.330
	21	7	7	3	0.330
	28	6	9	2	0.160
	60	2	13	2	1.000
	90	4	10	3	0.720

*significant

### Dimensions of the keratinized tissue

The mean graft width in the last evaluation was 6.47 ± 1.37 mm and 6.0 ± 1.27 mm in the study and control groups, respectively (p-value = 0.19; non-significant). The mean graft size was 80.88 ± 30.21 mm^2^ in the EPO side, and 71.35 ± 15.62 mm^2^ in the control side (p-value = 0.25; non-significant).

## Discussion

This study aimed to evaluate the effects of the topical application of erythropoietin on the outcome of free gingival graft performed to increase the keratinized tissue width. The results of this study showed that EPO could accelerate the rate of healing and reduce the degree of inflammation in free gingival grafts. The areas treated with EPO gel generally healed faster and experienced less inflammation compared to the control sites. Similar results have been reported in terms of improved wound healing and reduced inflammation using topical or systemic EPO in previous articles. In an animal study by Hamed et al. [24], topical application of erythropoietin accelerated the process of dermal wound healing in diabetic mice. Several other animal studies reported positive effects of EPO on ischemic [22], diabetic [27] and burn [32] wound healing.

In a clinical study, Yaghobee et al. [29] evaluated the effect of topical EPO (4000 IU/ml) on palatal wound healing after gingival graft harvesting on days 7, 14, 21, and 28 after surgery. Compared to the control group (vehicle gel), on the 21st day, the test group epithelialization was significantly better, and on the 28th day, with direct examination, it significantly improved healing with less inflammation present. At other time points or with the observation of photographs, there was no significant difference between the two groups.

Bader et al. [33] also reported that the use of erythropoietin could significantly accelerate the epithelialization and healing of wound. One of the cases in their clinical study had two skin graft donor sites of the same size, one of which was treated with EPO 3000 IE/hydrogel while the other site received hydrogel alone on days 0, 3, and 6. Seven days after surgery, the wound showed complete re-epithelialization on the EPO treated side, while incomplete healing was seen on the other side [33]. In an earlier study by Yaghobee et al. [29], the palatal wound epithelialization was completed in most EPO samples on day 21, which was significantly more than control group. The lower depth of skin lesion created compared to the palatal wound (0.3 versus 1.5 mm) and the greater frequency of EPO application could be the reason for the faster completion of epithelialization in the study.

The positive effects of EPO on wound healing is likely to be mediated by improved angiogenesis [25, 27, 32], increased synthesis of vascular endothelial growth factor (VEGF)‌ [24, 27, 32], and accelerated wound epithelialization [17]. Erythropoietin can also stimulate collagen production [22], and lower apoptosis has been spotted in EPO-treated wounds [24, 25]. Also erythropoietin is likely to play an indirect role in the wound healing process through the impact on blood coagulation [34, 35] and the immune system [37]. Therefore, more research is still needed to determine its exact function in the wound healing process in animal and human models.

Considering the results of this study on days 60 and 90, no significant difference in terms of tissue color match, contour, and texture between the test and control groups was found. This means that erythropoietin does not affect the final outcome of graft healing, but simply causes the graft to go through the healing process slightly faster. This, however, equates less pain and discomfort for the patient and sooner achievement of the desired result.

Based on direct and/or indirect examinations, the inflammation halo was significantly less evident around the test side compared to the control side at all timepoints, except for the 7th day, during which no significant difference was found between the groups. Although some degrees of inflammation are essential for the wound repair, excessive inflammatory response will delay healing [38]. According to Hamed et al. [25], topical erythropoietin reduces inflammatory cytokines TNF-α, IL-6, and IL-1β in diabetic rat wounds. Yaghobee et al. [29] also found that the palatal wound inflammation was less severe in the EPO group compared to the control group. However, this difference was statistically significant only on day 28.

Other growth factors, individually or in comparison to erythropoietin or in addition to it have been used in some studies. Curro et al. [11] pioneered to have reported a reduction in transglutaminase 1 in gingival tissues in the course of the periodontal disease. They concluded that TG1 may promote cell cohesion in gingival epithelium. Fayazzadeh et al. [39] compared the effects of subcutaneous injections of normal saline, erythropoietin, and fibroblast growth factor-2 (FGF-2) to prevent skin flap necrosis in rats. The resultant necrotic area in the EPO group was significantly smaller than the control group, but larger compared to the FGF-2 group. In another study, Hamed et al. [25] reported the cumulative effects of topical fibronectin and EPO to improve diabetic mice skin wound repair.

Sorg et al. [17] studied the effects of EPO administration dose on skin wound healing in mice. According to the results, repetitive injections of low doses and a single injection of high doses of EPO accelerates wound healing, but repetitive high doses of the drug will impair the healing process. Indeed, repeated high doses resulted in altered migration of the fibroblast and keratinocytes, and deteriorated vessel maturation.

Overall, the results of multiple studies including the present study, indicates that EPO has a considerable effect in wound healing. However, the reported effect size has been different which may have been influenced by the drug administration route and the circumstances of the area to be treated. For example, the presence of saliva and tongue movements in the oral cavity causes the drug to be washed away in a short time, and as a result the drug effects are limited compared to dermal applications. The dose and frequency of application in oral cavity, therefore, has to be specified to the region to gain optimum results of healing in gingival/mucosal wounds.

Due to the limited human trials regarding the effects of topical EPO, further research is still needed on different types of wounds (acute/chronic; small/large; various locations in body), with various administration routes, doses and frequencies. Also future research may focus on the synergistic effects of EPO with other growth factors needs to be investigated as well.

## Conclusions

Erythropoietin may accelerate healing and reduce inflammation in free gingival graft recipient sites, which could be related to improved angiogenesis and epithelialization mediated by this factor. However, it does not affect the final graft outcome regarding the size, contour, texture, and color match in short term. More investigation is needed to find the optimum dose and frequency of EPO application.

## Data Availability

The datasets used and/or analysed during the current study are available from the corresponding author on reasonable request.

## Abbreviations

EPO: Erythropoietin
FGG: Free gingival graft
